# Form factors of the isovector scalar current and the $\eta \pi$ scattering phase shifts

**DOI:** 10.1140/epjc/s10052-015-3715-z

**Published:** 2015-10-13

**Authors:** M. Albaladejo, B. Moussallam

**Affiliations:** Groupe de Physique Théorique, IPN (UMR8608), Université Paris-Sud 11, Orsay, France; Instituto de Física Corpuscular (IFIC), Centro Mixto CSIC-Universidad de Valencia, Valencia, Spain

## Abstract

A model for S-wave $\eta \pi$ scattering is proposed which could be realistic in an energy range from threshold up to above 1 GeV, where inelasticity is dominated by the $K{\bar{K}}$ channel. The *T*-matrix, satisfying two-channel unitarity, is given in a form which matches the chiral expansion results at order $p^4$ exactly for the $\eta \pi \rightarrow \eta \pi$, $\eta \pi \rightarrow K{\bar{K}}$ amplitudes and approximately for $K{\bar{K}}\rightarrow K{\bar{K}}$. It contains six phenomenological parameters. Asymptotic conditions are imposed which ensure a minimal solution of the Muskhelishvili–Omnès problem, thus allowing one to compute the $\eta \pi$ and $K{\bar{K}}$ form factor matrix elements of the $I=1$ scalar current from the *T*-matrix. The phenomenological parameters are determined such as to reproduce the experimental properties of the $a_0(980)$, $a_0(1450)$ resonances, as well as the chiral results of the $\eta \pi$ and $K{\bar{K}}$ scalar radii, which are predicted to be remarkably small at $O(p^4)$. This *T*-matrix model could be used for a unified treatment of the $\eta \pi$ final-state interaction problem in processes such as $\eta '\rightarrow \eta \pi \pi$, $\phi \rightarrow \eta \pi \gamma$, or the $\eta \pi$ initial-state interaction in $\eta \rightarrow 3\pi$.

## Introduction

The properties of the $\eta \pi$ scattering amplitude are much less known than those of $\pi \pi$ or $K\pi$ scattering. In the framework of three-flavour chiral symmetry (in which the $\eta$ is a pseudo-Goldstone boson) a specific prediction can be made that the $\eta \pi$ interaction should be considerably weaker than the $\pi \pi$ or $K\pi$ interactions [1] at low energies. This feature has not yet been verified either experimentally or in lattice QCD. It is possibly related to the apparent absence of a broad light $I=1$ scalar resonance.

A global description of $\pi \eta$ scattering (in particular of the elastic channel and the leading inelastic channel $\pi \eta \rightarrow K{\bar{K}}$) would enable one to perform a universal treatment of the final-sate (or initial-sate) interaction involving the $\pi \eta$ system. A particularly interesting application would be to the $\eta \rightarrow 3\pi$ amplitude. Precision measurements of these decay modes should be exploited in an optimal way for the determination of isospin violating quark mass ratios. For this purpose, it is necessary to combine chiral expansion expressions with general dispersive treatments of rescattering [2, 3]. An extension of these approaches to include $\eta \pi$ rescattering would allow one to take into account explicitly the $a_0$–$f_0$ “mixing” effect,^1^ which was claimed to be significant [5] for $\eta \rightarrow 3\pi$.

The available experimental information on $\eta \pi$ scattering have been derived via the final-state interaction effects in production processes and they concern, essentially, the properties of the resonances. The two prominent resonances which have been observed in the *S*-wave are the $a_0(980)$ and the $a_0(1450)$. We wish to address here the problem of determining more global properties of the *S*-wave amplitude, i.e., the determination of phase shifts and inelasticities in the small- to medium-energy range such as to be compatible with the properties of the resonances and also obey further theoretical constraints.

Several models of the $\eta \pi$*S*-wave scattering amplitude have been proposed in the literature [6–9]. Our approach enforces a correct matching with the chiral expansion of the amplitudes at low energy in a way somewhat similar to Refs. [6, 7]. In addition, we propose here to consider the form factor $F_S^{\eta \pi }$ (and $F_S^{K{\bar{K}}}$) associated with the scalar isovector current operator ${\bar{u}}d$, in parallel with the *T*-matrix. Form factors are the simplest quantities to which analyticity based final-state interaction methods can be applied. We will follow the same general method as was proposed for the scalar isoscalar pion (and kaon) form factors [10] and proved capable of determining the scalar radius of the pion ${\langle r^2 \rangle }_S^{\pi \pi }$ rather accurately (see Refs. [10–16] for theoretical calculations, and Refs. [17–19] for lattice determinations). Its application to the strangeness changing $K\pi$ scalar form factor and the corresponding scalar radius ${\langle r^2 \rangle }_S^{K\pi }$ were discussed in Refs. [20–22]. Form factors are constrained by chiral symmetry at low energy and, even though the convergence of the three-flavour chiral expansion may be rather slow, one still expects correct order of magnitudes to be provided at order $p^4$. At this order, a simple relation between the $\eta \pi$ and the $K\pi$ scalar radii is predicted,

1 $$\begin{aligned} \left. \dfrac{{\langle r^2 \rangle }_S^{\eta \pi }}{{\langle r^2 \rangle }_S^{K\pi }}\right| _{p^4} = 0.52\pm 0.02. \end{aligned}$$

This relation implies that the $$\eta \pi $$ radius is remarkably small $${\langle r^2 \rangle }_S^{\eta \pi }\simeq 0.1$$$$\hbox {fm}^2$$. We will show that this result provides a stringent constraint on the determination of the phase shifts and inelasticities.

The plan of the paper is as follows. We start with the chiral perturbation theory (ChPT) expansions of the scalar form factors $F_S^{\eta \pi }$, $F_S^{K{\bar{K}}}$ and with the $\eta \pi$ and $K{\bar{K}}$ scattering amplitudes at next-to-leading order (NLO). Next, we recall the general dispersive integral equations from which one can compute the form factors starting from a given *T*-matrix, provided suitable asymptotic conditions are imposed. We then describe our chiral *K*-matrix type model for the *T*-matrix, which involves six phenomenological parameters. It is designed such that, at low energies, the contributions involving these parameters have chiral order $p^6$ (that is, NNLO) and that a proper matching with the ChPT expressions at NLO holds except, however, for the $K{\bar{K}}\rightarrow K{\bar{K}}$ amplitude, for which the matching is only approximate. Finally, the determination of the phenomenological parameters is discussed so as to satisfy the experimental constraints on the $a_0$ resonances and the chiral constraints on the scalar form factors.

## ChPT expansions of $\eta \pi ^+$, ${\bar{K}}^0K^+$ form factors and scattering amplitudes

### Form factors and scalar radii

Let us introduce the following two form factors associated with the isospin one charged scalar operator ${\bar{u}}d$:

2 $$\begin{aligned} B_0\,F_S^{\eta \pi }(s)= & {} {\langle \eta (p_1)\pi ^+(p_2)\vert {\bar{u}}d(0) \vert 0 \rangle },\nonumber \\ B_0\,F_S^{K{\bar{K}}}(s)= & {} {\langle {{\bar{K}}^0}(p_1){K^+}(p_2) \vert {\bar{u}}d(0) \vert 0 \rangle }, \end{aligned}$$

where $s=(p_1+p_2)^2$. We have computed these form factors at next-to-leading order (NLO) in the chiral expansion. The detailed expressions are given in Appendix A. From Eqs. (A.2) and (A.3) in that appendix, it is easy to derive the expressions of the scalar radii, which are defined as

3 $$\begin{aligned} {\langle r^2 \rangle }_S^{PQ}= 6{\dot{F}_S^{PQ}(0)}/{F_S^{PQ}(0)}. \end{aligned}$$

For $\eta \pi$ and $K{\bar{K}}$ one obtains

4 $$\begin{aligned} {\langle r^2 \rangle }_S^{\eta \pi }= & {} \dfrac{6}{F_\pi ^2}\left[ 4\,L^r_{5} + \dfrac{1}{16\pi ^2}\,\left( -\dfrac{3}{4}L_K -\dfrac{11}{12}\right) \right. \nonumber \\&\left. + \dfrac{m_\pi ^2}{3}\,{\bar{J}}'_{\pi \eta }(0)\right] \end{aligned}$$

5 $$\begin{aligned} {\langle r^2 \rangle }_S^{K{\bar{K}}}= & {} \dfrac{6}{F_\pi ^2}\left[ 4\,L^r_{5} + \dfrac{1}{16\pi ^2}\left( -\dfrac{1}{2}\,L_\eta - \dfrac{1}{4}\,L_K\right. \right. \nonumber \\&\left. \left. -\dfrac{1}{2}\,R_{\pi \eta } -\dfrac{1}{4}\right) \ -\dfrac{2m_K^2}{3}\,{\bar{J}}'_{\pi \eta }(0)\right] , \end{aligned}$$

where $L_P$, $R_{PQ}$ are logarithmic functions of the pseudo-scalar meson masses,

6 $$\begin{aligned} L_P=\log \frac{m_P^2}{\mu ^2},\quad R_{PQ}=\frac{m_P^2\log (m_P^2/m_Q^2)}{m_P^2-m_Q^2}, \end{aligned}$$

with $\mu$ a renormalisation scale. These scalar radii depend on only one of the Gasser–Leutwyler coupling constants [23], $L^r_5$. It is instructive to compare them with the analogous $K\pi$ scalar radius associated with the strangeness changing scalar current, which also depends only on $L^r_5$ [24],

7 $$\begin{aligned} {\langle r^2 \rangle }_S^{K\pi }&= \dfrac{6}{F_\pi ^2}\left[ 4\,L^r_{5} -\dfrac{1}{8}\dfrac{1}{16\pi ^2}\left( 6L_K +5R_{\pi K}+ R_{\eta K} \right) \right] \nonumber \\&\quad +\delta _2. \end{aligned}$$

The explicit expression of $\delta _2$, from Ref. [24], is reproduced in Appendix A. One remarks that the three scalar radii ${\langle r^2 \rangle }_S^{\eta \pi }$, ${\langle r^2 \rangle }_S^{K{\bar{K}}}$, ${\langle r^2 \rangle }_S^{K\pi }$ have exactly the same dependence on the coupling $L^r_5$, which means that they should be equal in the large $N_c$ limit of QCD. In reality, they are rather different. Using e.g. $L^r_5=(1.23\pm 0.06)\cdot 10^{-3}$ (from Ref. [25], see Sect. 2.3 below) one finds^2^ for $\eta \pi$ and $K{\bar{K}}$

8 $$\begin{aligned} {\langle r^2 \rangle }^{\eta \pi }_S= & {} 0.092\pm 0.007\ \hbox {fm}^2,\nonumber \\ {\langle r^2 \rangle }^{K{\bar{K}}}_S= & {} 0.136\pm 0.007\ \hbox {fm}^2, \end{aligned}$$

while for $K\pi$ one finds

9 $$\begin{aligned} {\langle r^2 \rangle }^{K\pi }_S = 0.177 \pm 0.007~\hbox {fm}^2. \end{aligned}$$

This shows that the $\eta \pi$ scalar radius is suppressed by a factor of 2 as compared to the $K\pi$ scalar radius.

### Scattering amplitudes at $O(p^4)$

We consider the three scattering amplitudes involving the $\eta \pi ^+$ and the ${{\bar{K}}^0}{K^+}$ channels and we label the $\eta \pi ^+$ channel as 1 and the ${{\bar{K}}^0}{K^+}$ channel as 2. At chiral order $p^2$ the amplitudes read,

10 $$\begin{aligned} {\mathcal T}^{11}_{(2)}(s,t,u)= & {} \dfrac{m_\pi ^2}{3F_\pi ^2}\nonumber \\ {\mathcal T}^{12}_{(2)}(s,t,u)= & {} \dfrac{\sqrt{6}}{12F_\pi ^2}\,(3s-4m_K^2)\\ {\mathcal T}^{22}_{(2)}(s,t,u)= & {} \dfrac{1}{4F_\pi ^2}(s+(t-u)).\nonumber \end{aligned}$$

The corrections of chiral order $p^4$ to these amplitudes can be expressed in terms of a set of functions of one variable, analytic with a right-hand cut, according to the so-called reconstruction theorem [26] (see also the review [27]),

11 $$\begin{aligned} {\mathcal T}^{11}_{(4)}(s,t,u)= & {} U_0^{11}(s)+U_0^{11}(u)+W_0^{11}(t),\nonumber \\ {\mathcal T}^{12}_{(4)}(s,t,u)= & {} U_0^{12}(s)+[W_0^{12}(t)+(s-u) W_1(t) \nonumber \\&+(t\leftrightarrow u)]\\ {\mathcal T}^{22}_{(4)}(s,t,u)= & {} U_0^{22}(s)+(t-u)U_1(s)+V_0(t)\nonumber \\&+(s-u)V_1(t)+W_0^{22}(u).\nonumber \end{aligned}$$

The detailed expressions of the functions $U_0^{ab}$, $W_0^{ab}$, $U_j$, $V_j$ are given in Appendix B. The resulting amplitudes are equivalent to previous calculations [1, 7]. We define the partial-wave amplitudes as

12 $$\begin{aligned} T_J^{ab}(s)=\frac{1}{32\pi }\int _{-1}^1 {\mathcal T}^{ab}(s,t(z^{ab}),u(z^{ab}))\,\mathrm{d}z^{ab} \end{aligned}$$

such that the unitarity relation, in matrix form, reads

13 $$\begin{aligned} \mathrm{Im\,}\varvec{T}_J(s) = \varvec{T}_J(s)\,\varvec{\varSigma }(s) \, \varvec{T}^\dagger _J(s) =\varvec{T}_J^\dagger (s)\,\varvec{\varSigma }(s) \, \varvec{T}_J(s) \end{aligned}$$

with

14 $$\begin{aligned} \varvec{\varSigma }(s)=\left( \begin{array}{l@{\quad }l} \sigma _1(s)\theta (s-(m_\eta +m_\pi )^2) &{} 0\\ 0 &{} \sigma _2(s)\theta (s-4m_K^2) \\ \end{array}\right) ,\nonumber \\ \end{aligned}$$

and

15 $$\begin{aligned}&\sigma _1(s)=\frac{\sqrt{\lambda _{\eta \pi }(s)}}{s},\quad \sigma _2(s)=\sqrt{\frac{s-4m_K^2}{s} },\nonumber \\&\lambda _{\eta \pi }(s)=(s-(m_\eta -m_\pi )^2)(s-(m_\eta +m_\pi )^2). \end{aligned}$$

The relation between the partial wave *S*- and *T*-matrices then reads

16 $$\begin{aligned} \varvec{S}_J(s)=1 +2i \sqrt{\varvec{\varSigma }(s)}\, \varvec{T}_J(s)\,\sqrt{\varvec{\varSigma }(s)}. \end{aligned}$$

In Eq. (12), $z^{ab}$ designate the cosines of the centre-of-mass scattering angles, which are related to the Mandelstam variables by

17 $$\begin{aligned} t,u(z^{11})= & {} \frac{1}{2}\left( 2m_\eta ^2+2m_\pi ^2-s \pm \dfrac{\lambda _{\eta \pi }(s) z^{11}-\varDelta _{\eta \pi }^2}{s} \right) ,\nonumber \\ t,u(z^{12})= & {} \frac{1}{2}\left( m_\eta ^2+m_\pi ^2+2m_K^2-s \pm \sqrt{\lambda _{\eta \pi }(s)}\,\sigma _2(s)\,z^{12}\right) ,\nonumber \\ t,u(z^{22})= & {} \frac{1}{2}(4m_K^2-s)(1\mp z^{22}), \end{aligned}$$

with $\varDelta _{\eta \pi }=m_\eta ^2-m_\pi ^2$. The first two of these relations become singular when $s\rightarrow 0$. This implies that the chiral expansions of the $\eta \pi \rightarrow \eta \pi$ and $\eta \pi \rightarrow K{\bar{K}}$ partial-wave amplitudes become invalid when *s* is too close to zero. If we assume a domain of validity for the expansion of the unprojected amplitudes when $|s|,\, |t|,\, |u| \,{ \lesssim }\, 0.5~\hbox {GeV}^2$, then the chiral expansions of the partial-wave amplitudes $T^{11}_J$, $T^{12}_J$ should converge with *s* lying in the range $0.17 \,{ \lesssim }\, s \,{ \lesssim }\, 0.5~\hbox {GeV}^2$ and $0.05 \,{ \lesssim }\, s \,{ \lesssim }\, 0.5~\hbox {GeV}^2$ respectively.

From now on, we will consider only the $J=0$ partial wave and will drop the *J* subscript. With the subscript now indicating the chiral order, the $J=0$ partial-wave amplitudes at $O(p^2)$ are simply derived from (10)

18 $$\begin{aligned}&T^{11}_{(2)}(s)=\frac{1}{16\pi }\frac{m_\pi ^2}{3F_\pi ^2},\quad T^{12}_{(2)}(s)=\frac{1}{16\pi }\frac{\sqrt{6}(3\,s-4\,m_K^2)}{12F_\pi ^2},\nonumber \\&T^{22}_{(2)}(s)=\frac{1}{16\pi }\frac{s}{4F_\pi ^2} . \end{aligned}$$

The corrections of chiral order $p^4$ to these $J=0$ partial-wave amplitudes can be written as

19 $$\begin{aligned} T^{ij}_{(4)}(s)=\dfrac{1}{16\pi }\left( U_0^{ij}(s) +{\hat{U}}_0^{ij}(s)\right) \end{aligned}$$

where

20 $$\begin{aligned}&{\hat{U}}_0^{11}(s)=\frac{1}{2}\int _{-1}^1 \mathrm{d}z^{11}\, (U_0^{11}(u) + W_0^{11}(t))\nonumber \\&{\hat{U}}_0^{12}(s)= \int _{-1}^1 \mathrm{d}z^{12}\, (W_0^{12}(t)+(s-u)\,W_1(t))\\&{\hat{U}}_0^{22}(s)= \frac{1}{2}\int _{-1}^1 \mathrm{d}z^{22}\, (V_0(t)+ (s-u)\,V_1(t) +W_0^{22}(u))\nonumber \end{aligned}$$

The functions ${\hat{U}}_0^{ij}(s)$ carry the left-hand cuts of the partial-wave amplitudes $T^{ij}$. These cuts are as follows [28]:

$$T^{11}$$:
A real cut on $[-\infty ,(m_\eta -m_\pi )^2]$ and a complex circular cut centred at $s=0$ with radius $\varDelta _{\eta \pi }$.
$$T^{12}$$:
A real cut on $[-\infty ,0]$ and a complex quasi-circular cut which intersects the real axis at $-\varDelta _{\eta \pi } m_K/(m_K+m_\eta )$ and $\varDelta _{\eta \pi } m_K/(m_K+m_\pi )$.
$$T^{22}$$:
A real cut on $[-\infty ,4m_K^2-4m_\pi ^2]$.

As a final remark, at NLO, each one of the functions $U_0^{ij}$, $W_0^{ij}$, $U_1$, $V_j$ can be written as the sum of a polynomial part and one involving a combination of functions ${\bar{J}}_{PQ}$ (see Appendix B). The latter part is constrained by unitarity. For instance, for the functions $U_0^{ij}$, one can write, in matrix form,

21 $$\begin{aligned} \varvec{U_0}(s)= & {} \varvec{P_0}(s)+ (16\pi )^2 \,\varvec{T}_{(2)}(s)\nonumber \\&\times \left( \begin{array}{l@{\quad }l} {\bar{J}}_{\pi \eta }(s) &{} 0 \\ 0 &{} {\bar{J}}_{K {\bar{K}}}(s) \end{array}\right) \varvec{T}_{(2)}(s). \end{aligned}$$

### Influence of the $1/N_c$ suppressed couplings

**Table 1:** Two sets of central values of $L_i^r(\mu )$ (${\times } 10^{3}$) with $\mu =0.77$ GeV, from the NLO fits performed Ref. [25]

$10^3\times$	$L_1^r$	$L_2^r$	$L_3^r$	$L_4^r$	$L_5^r$	$L_6^r$	$L_7^r$	$L_8^r$
(A)	1.11	1.05	$-$3.82	1.87	1.22	1.46	$-$0.39	0.65
(B)	1.00	1.48	$-$3.82	0.30	1.23	0.14	$-$0.27	0.55

**Fig. 1:**
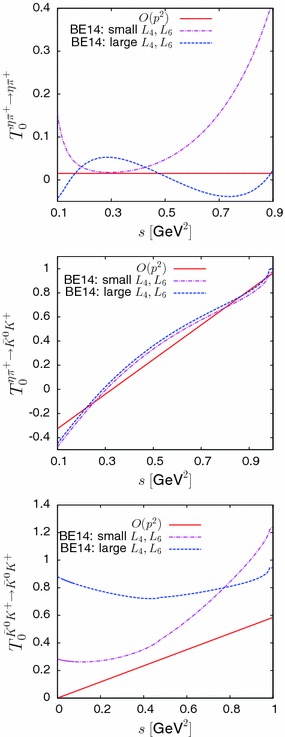
Real parts of the three $J=0$ partial-wave amplitudes $\eta \pi ^+\rightarrow \eta \pi ^+$, $\eta \pi ^+\rightarrow {{\bar{K}}^0}{K^+}$ and ${{\bar{K}}^0}{K^+}\rightarrow {{\bar{K}}^0}{K^+}$ at leading and next-to-leading order in ChPT

The values of the low-energy couplings (LEC’s) $$L_i^r$$, $$i=1\cdots 8 $$, are needed in order to evaluate numerically the chiral amplitudes. A recent update of the values of the couplings $$L_i^r$$ has been presented in Ref. [25] based on global fits involving a number of low-energy observables. We reproduce in Table 1 two sets of values which correspond to NLO expansions (which seem appropriate here since we are using NLO formulae). The set labelled (A) in Table 1 corresponds to an unconstrained fit and it leads to rather large values of the couplings $$L_4$$, $$L_6$$ and $$L_2-2L_1$$, which are suppressed in the large $$N_c$$ limit [23]. The set (B) in the table corresponds to a fit which is constrained to enforce compatibility with the results from lattice QCD simulations on $$L_4^r$$ and $$L_6^r$$. We will consider it to be more plausible, since the strong deviations from the large $$N_c$$ limit are possibly an artefact of attempting to reproduce certain observables which are sensitive to NNLO rescattering effects (like the $$I=J=0$$$$\pi \pi $$ scattering length) using NLO formulae. Figure 1 illustrates the sensitivity of the $$I=1$$ amplitudes considered here to the $$1/N_c$$ suppressed couplings. The shape of the $$\eta \pi \rightarrow \eta \pi $$ amplitude is quite different if one uses the set (A) or the set (B). This is also reflected in the values of the $$J=0$$ threshold parameters. Defining the scattering length $$a_0$$ and the scattering range $$b_0$$ as in Ref. [1],

22 $$\begin{aligned} \frac{2}{\sqrt{s}}\,T^{11}(s)= a_0 + b_0\, p^2 +\cdots \end{aligned}$$

with $\sqrt{s}=\sqrt{m_\pi ^2+p^2}+\sqrt{m_\eta ^2+p^2}$, one finds

23 $$\begin{aligned}&m_\pi \,a_0 = 6.7\times 10^{-3},\nonumber \\&\quad m_\pi \,b_0 = -15.0\times 10^{-3} \quad (\hbox {Large }L_4,L_6) \nonumber \\&m_\pi \,a_0 = 16.2\times 10^{-3},\nonumber \\&\quad m_\pi \,b_0 = 10.6\times 10^{-3} \quad (\hbox {Small }L_4,L_6). \end{aligned}$$

The two sets of couplings thus lead to rather different values of the scattering length $a_0$ while the values of the scattering range $b_0$ differ in their sign. At leading chiral order, one has $m_\pi \,a_0 = 6.2\times 10^{-3}$, $b_0=0$. At NLO, a low-energy theorem (LET) for $a_0$ was derived in Ref. [29], in the form of a linear relation

24 $$\begin{aligned} \left. a_{0}\right| _{\mathrm{NLO}} = \lambda \, \left. a_{0,\pi \pi }^2\right| _{\mathrm{NLO}} +\mu \end{aligned}$$

where $a_{0,\pi \pi }^2$ is the $\pi \pi$ scattering length with $J=0$, $I=2$ and $\lambda$, $\mu$ are simple functions of the masses $m_\pi$, $m_K$, $m_\eta$ and the decay constants $F_\pi$, $F_K$. The most precise determinations of the *S*-wave $\pi \pi$ scattering lengths are based on Roy equations solutions. Using the values quoted in two recent analysis of these equations [14, 30] in the LET relation (24), one obtains

25 $$\begin{aligned}&a_{0,\pi \pi }^2=-0.0444\pm 0.0010\;(\hbox {ref.}\, [14])\nonumber \\&\quad \longrightarrow a_0=(-0.22\pm 6.26)\times 10^{-3}\nonumber \\&a_{0,\pi \pi }^2=-0.042\pm 0.0040\;(\hbox {ref.}\, [30])\nonumber \\&\quad \longrightarrow a_0=(14.8\pm 25.0)\times 10^{-3}. \end{aligned}$$

This illustrates that the LET is practically useful only if $a_{0,\pi \pi }^2$ is known to a very high precision. The result of Ref. [14] is associated with a rather small error of $2.5~\%$. However, the result derived from the Roy equations concerns the physical value of the scattering length rather than the NLO value which enters into the LET. An additional error should therefore be introduced in Eq. (25) in order to account for the difference $a_{0,\pi \pi }^2-a_{0,\pi \pi }^2\vert _{\mathrm{NLO}}$, which could easily be as large than $5~\%$. This observation then limits the effectiveness of the LET for determining $a_0$.

The ${{\bar{K}}^0}{K^+}\rightarrow {{\bar{K}}^0}{K^+}$ partial-wave amplitude vanishes at $s=0$ at leading chiral order (18). This zero, however, is accidental since it is not associated with a soft pion theorem. Figure 1 shows that, indeed, the NLO corrections are substantial. The corrections corresponding to the $L_i$ set (B), with small $1/N_c$ violations, have a more reasonable size than those from set (A). The amplitude $\eta \pi ^+\rightarrow {{\bar{K}}^0}{K^+}$ has a zero at $s=4m_K^2/3$ at $O(p^2)$ which corresponds to a soft pion Adler zero. Figure 1 shows that the NLO corrections are rather small in this case and that there is little difference between the couplings of set (A) and set (B).

## Form factors from dispersive integral equations

We follow here a general approach to the construction of form factors which implements unitarity relations and chiral constraints and, additionally, impose the absence of zeros and consistency with the QCD asymptotic behaviour. We will briefly review this method below, which was applied previously to the scalar $\pi \pi$ and $\pi K$ form factors [10, 20], and allows one to relate the form factors and the corresponding *S*-wave scattering amplitudes via a set of integral equations. The $I=1$ scalar form factors $F_S^{\eta \pi }$, $F_S^{K{\bar{K}}}$, which we will discuss here, were considered previously in Ref. [31]. The approach followed in Ref. [31] differs from ours in that the constraints on the zeros and the asymptotic behaviour were not imposed.

### Phase dispersive representation

The crucial property of two-meson form factors is that they can be defined as analytic functions in the complex energy plane, with a cut lying on the positive real axis in the range $s > (m_P+m_Q)^2$ [32]. In the asymptotic region, $|s|\rightarrow \infty$, the general arguments concerning exclusive processes in QCD [33] predict that a two-meson scalar form factor $F_S$ should obey a power law behaviour,

26 $$\begin{aligned} F_S(s)\vert _{s\rightarrow \infty } \sim 1/s \end{aligned}$$

up to logarithms. Making the assumption that the form factor $F_S$ has no zeros in the complex plane, one can derive a minimal phase dispersive representation (e.g. [34]),

27 $$\begin{aligned} F_S(s)=F_S(0)\,\exp \left[ \frac{s}{\pi }\int _{s_0}^\infty \frac{\phi _S(s')}{s'\,(s'-s)}\,\mathrm{d}s'\right] , \end{aligned}$$

where the phase is defined from $F(s+i\epsilon )=|F_s(s)|\hbox {e}^{i\phi _S(s)}$. The QCD asymptotic behaviour (26) is reproduced from Eq. (27) provided that the phase has the asymptotic limit:

28 $$\begin{aligned} \lim _{s'\rightarrow +\infty }\phi _S(s')=\pi . \end{aligned}$$

The scalar radius, finally, is given by a simple integral as a function of $\phi _S$,

29 $$\begin{aligned} {\langle r^2 \rangle }_S= \frac{6}{\pi }\int _{s_0}^\infty \frac{\phi _S(s')}{(s')^2}\,\,\mathrm{d}s'. \end{aligned}$$

If *n* complex zeros were present, then the right-hand side of Eq. (27) would have to be multiplied by a polynomial of degree *n* and the asymptotic phase would have to be $(n+1)\pi$. The minimality assumption is equivalent to stating that the increase of the phase in the energy region $\sqrt{s} >2$ GeV should be less than $\pi$. This is plausible since no sharp resonances are present in this region.

### Determination of the form factors from the *T*-matrix

As emphasised in Ref. [15], these phase relations are of particular interest for those form factors which involve at least one pion, $F_S^{\pi P}$ with $P=\pi$, *K* or $\eta$, which interests us here. This is simply because the scattering amplitudes $\pi P\rightarrow \pi P$ are *elastic* in a finite low-energy region. In this region, the form factor phase $\phi _S^{\pi P}$ is constrained from Watson’s theorem to be exactly equal to the elastic scattering phase shift. The energy region in which inelasticity can be neglected to a good approximation extends up to the $K{\bar{K}}$ threshold for $\pi \pi$ and we expect the same property to hold also^3^ for $\pi \eta$. The asymptotic value of the form factor phase is also known and one may estimate that $\phi _S^{\pi P}$ should be smoothly approaching its asymptotic value when $\sqrt{s}\,{ \gtrsim }\, 2$ GeV. There only remains to determine $\phi _S^{\pi P}$ in the intermediate-energy region, that is, in the case of $\eta \pi$, in the region $1\le \sqrt{s}\,{ \lesssim }\, 2$ GeV. In this region, we further expect that the fastest energy variation should take place close to 1 GeV, associated with the sharp onset of inelasticity triggered by the presence of the $a_0(980)$ resonance which is known to couple strongly to the $K{\bar{K}}$ channel [35]. This suggests to consider a framework which takes into account only the dominant inelastic channel and ignores all the other ones. In this case, the two form factors $F_S^{\eta \pi }$, $F_S^{K{\bar{K}}}$ obey a closed set of Muskhelishvili–Omnès coupled integral equations,

30 $$\begin{aligned}&\left( \begin{array}{l} F_S^{\eta \pi }(s)\\ F_S^{K{\bar{K}}}(s)\\ \end{array}\right) = \dfrac{1}{\pi }\int _{(m_\eta +m_\pi )^2}^\infty \dfrac{\mathrm{d}s'}{s'-s} \left( \begin{array}{l@{\quad }l} T^{11}(s) &{} T^{12}(s) \\ T^{12}(s) &{} T^{22}(s) \\ \end{array}\right) ^*\nonumber \\&\quad \times \left( \begin{array}{l} \sigma _1(s')\,F_S^{\eta \pi }(s')\\ \sigma _2(s')\,F_S^{K{\bar{K}}}(s')\theta (s'-4m_K^2)\\ \end{array}\right) . \end{aligned}$$

These equations encode the property of analyticity of the form factors, the asymptotic behaviour (which allows for an unsubtracted dispersive representation) and two-channel unitarity. One can express the two-channel *S*-matrix in terms of two phase shifts and one inelasticity parameter in the usual way,

31 $$\begin{aligned}&\varvec{S}=\left( \begin{array}{l@{\quad }l} \eta \,\hbox {e}^{2i\delta _{11}} &{} i\sqrt{1-\eta ^2}\, \hbox {e}^{i(\delta _{11}+\delta _{22})} \\ i\sqrt{1-\eta ^2}\, \hbox {e}^{i(\delta _{11}+\delta _{22})} &{} \eta \,\hbox {e}^{2i\delta _{22}} \end{array}\right) ,\nonumber \\&\qquad 0\le \eta \le 1. \end{aligned}$$

We assume the following asymptotic conditions on the *S*-matrix parameters:

32 $$\begin{aligned} \lim _{s\rightarrow \infty } \eta (s)=1,\quad \lim _{s\rightarrow \infty } \delta _{11}(s)+\delta _{22}(s)=2\pi , \end{aligned}$$

which ensure that the so-called Noether index [36] (see also [37]) associated with the set of singular integral equations (30) is equal to 2. This, in general, implies that a unique solution is obtained once two arbitrary conditions are specified, for instance the values at $s=0$: $F_S^{\eta \pi }(0)$, $F_S^{K{\bar{K}}}(0)$, and that the solution form factors behave asymptotically as 1 / *s* [37].

In summary, solving the set of Eq. (30) for the form factors $F_S^{\eta \pi }$, $F_S^{K{\bar{K}}}$, one obtains a phase $\phi _S^{\eta \pi }$ which correctly matches with both the low- and the high-energy limits expectations and provides an interpolating model in the intermediate-energy region. The phase $\phi _S^{K{\bar{K}}}$ is also presented. In this case, however, there is no constraint from Watson’s theorem at low energy. One expects that the form factor $F_S^{K{\bar{K}}}$ will be more sensitive than $F_S^{\eta \pi }$ to the influence of the neglected inelastic channels.

More generally, one can use the system of equations (30) to define the Omnès matrix $\varOmega ^{ij}(s)$, which generalises the usual Omnès function [38]. Such a generalisation was first discussed in the case of $\pi \pi -K{\bar{K}}$ scattering in Refs. [39, 40]. The first column of the Omnès matrix is obtained by solving the system with the boundary conditions $\varOmega ^{11}(0)=1$, $\varOmega ^{21}(0)=0$ and the second column by solving with the conditions $\varOmega ^{12}(0)=0$, $\varOmega ^{22}(0)=1$ (see in Ref. [13] an appropriate numerical method for solving the linear system). The Omnès matrix allows one to treat the final-state interaction problem taking into account inelastic rescattering. For instance, one can express the $I=1$ scalar form factors in terms of the $\varvec{\varOmega }$ matrix,

33 $$\begin{aligned} \left( \begin{array}{l} F_S^{\eta \pi }(s)\\ F_S^{K{\bar{K}}}(s)\\ \end{array}\right) = \left( \begin{array}{l@{\quad }l} \varOmega ^{11}(s) &{} \varOmega ^{12}(s)\\ \varOmega ^{21}(s) &{} \varOmega ^{22}(s)\\ \end{array}\right) \left( \begin{array}{l} F_S^{\eta \pi }(0)\\ F_S^{K{\bar{K}}}(0)\\ \end{array}\right) . \end{aligned}$$

## Two-channel unitary *T*-matrix parametrisation with chiral matching

We seek a parametrisation of the $J=0$*T*-matrix which: (a) should satisfy exact elastic unitarity below the $K{\bar{K}}$ threshold and exact two-channel unitarity above, (b) should correctly match with ChPT for small values of *s*, i.e.,

34 $$\begin{aligned} T^{ij}(s) - (T^{ij}_{(2)}(s)+ T^{ij}_{(4)}(s)) =O(p^6) \end{aligned}$$

and (c) should be reasonably simple and flexible and be able to describe scattering in the low- to medium-energy region up to, say $$\sqrt{s}\simeq 2$$ GeV. We choose a representation somewhat similar to that proposed in Ref. [41] to describe $$J=0$$$$\pi K$$ scattering, belonging to the family of “unitary chiral” approaches. Such approaches were proposed, in the context of ChPT, firstly in Refs. [42, 43] and multichannel extensions were discussed in Refs. [44, 45] (we refer to the review [46] for a survey and a complete list of references). There are, however, some drawbacks to these methods. Poles can occur on physical sheets and, furthermore, the structure of the left-hand cuts is not quite correct. In particular, the left-hand cut of the chiral $$K{\bar{K}}\rightarrow K{\bar{K}}$$ amplitude $$T^{22}_{(4)}(s)$$, which extends up to $$s=4(m_K^2-m_\pi ^2)$$ is propagated to the amplitude $$T^{11}$$, via the unitarisation method, which actually spoils the unitarity of $$T^{11}$$ in the elastic region. While the resulting unitarity violation is numerically small [7, 47], we will prefer here to maintain exact unitarity at the price of relaxing the matching condition for the component $$T^{22}$$.

We start from a *K*-matrix type representation for the two-channel *T*-matrix

35 $$\begin{aligned} \varvec{T}(s)=(1- \varvec{K}(s)\varvec{\varPhi }(s))^{-1} \varvec{K}(s). \end{aligned}$$

This form is compatible with the symmetry of the *T*-matrix ($^t\varvec{T}=\varvec{T}$) provided both $\varvec{K}$ and $\varvec{\varPhi }$ are symmetric matrices. The matrix $\varvec{\varPhi }(s)$ must also satisfy

36 $$\begin{aligned}&\mathrm{Im\,}[\varvec{\varPhi }(s)]\nonumber \\&\quad =\left( \begin{array}{l@{\quad }l} \theta (s-(m_\eta +m_\pi )^2) \sigma _1(s) &{} 0 \\ 0 &{} \theta (s-4m_K^2) \sigma _2(s) \end{array}\right) ,\nonumber \\ \end{aligned}$$

which ensures that the *T*-matrix obeys the unitarity condition, provided that the matrix $\varvec{K}(s)$ remains real in the range $(m_\eta +m_\pi )^2\le s <\infty$. We take a representation of $\varvec{\varPhi }(s)$, satisfying Eq. (36), which is diagonal and contains four phenomenological parameters,

37 $$\begin{aligned}&\varvec{\varPhi }(s)\nonumber \\&\quad =\left( \begin{array}{l@{\quad }l} \alpha _1+\beta _1 s + 16\pi {\bar{J}}_{\eta \pi }(s) &{} 0 \\ 0 &{} \alpha _2+\beta _2 s + 16\pi {\bar{J}}_{K{\bar{K}}}(s) \end{array}\right) .\nonumber \\ \end{aligned}$$

The parameters $\alpha _i$, $\beta _i$ are assumed to be *O*(1) in the chiral counting. The *K*-matrix is written in terms of components with a definite chiral order,

38 $$\begin{aligned} \varvec{K}(s)=\varvec{K}_{(2)}(s)+{\varvec{K}}_{(4)}(s)+ \varvec{K}_{(6)}(s) \end{aligned}$$

where, as before, the subscript denotes the chiral order. In order to satisfy the matching condition (34) one must have

39 $$\begin{aligned}&\varvec{K}_{(2)}(s)= \varvec{T}_{(2)}(s),\nonumber \\&\varvec{T}_{(4)}(s)= {\varvec{K}}_{(4)}(s) +\varvec{T}_{(2)}(s) \varvec{\varPhi }_{(0)}(s)\varvec{T}_{(2)}(s). \end{aligned}$$

One can then express ${\varvec{K}}_{(4)}$ in terms of the polynomial and left-cut functions defined from Eqs. (19)–(21) (see also Appendix B),

40 $$\begin{aligned} {\varvec{K}}_{(4)}(s)= & {} \frac{1}{16\pi }\,(\varvec{P}_0(s) + \hat{\varvec{U}}_0(s))\nonumber \\&-\varvec{T}_{(2)}(s)\left( \begin{array}{l@{\quad }l} \alpha _1 &{}0 \\ 0 &{} \alpha _2\\ \end{array}\right) \varvec{T}_{(2)}(s). \end{aligned}$$

**Fig. 2:**
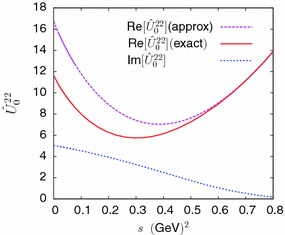
Comparison of the real part of function ${\hat{U}}_0^{22}$ with the approximation used in the unitary representation (35). Also shown is the imaginary part of ${\hat{U}}_0^{22}$

As explained above, we must use an approximation to the function ${\hat{U}}_0^{22}$ which has no cut on the real axis in the range $s \ge (m_\eta +m_\pi )^2$. This may be done by removing the parts which are proportional ${\bar{J}}_{\pi \pi }(t)$ and ${\bar{J}}_{\eta \pi }(t)$ (see Eq. (B.8B.9)) from the two functions $V_0(t)$ and $V_1(t)$, which appear in the angular integral which gives ${\hat{U}}_0^{22}$ (see Eq. (20)). Figure 2 compares this approximation of ${\hat{U}}_0^{22}$ to the exact function.

**Fig. 3:**
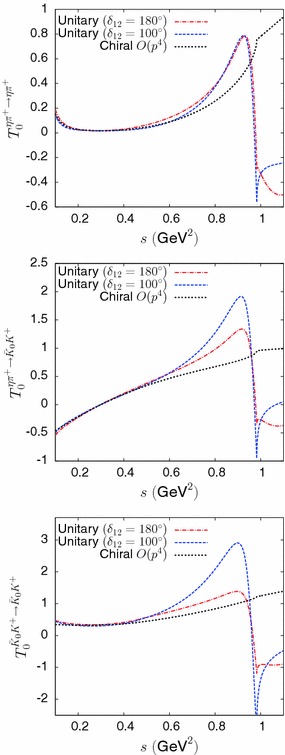
Comparison of the real parts of unitary partial-wave amplitudes $T^{ij}$ given from Eq. (35) and the corresponding chiral amplitudes at NLO

Finally, $\varvec{K}_{(6)}(s)$ is taken to be a pole term with the $O(p^4)$ part removed,

41 $$\begin{aligned} {K}^{ij}_{(6)}(s)= \frac{g_i g_j}{16\pi }\left( \frac{1}{m_8^2-s} - \frac{1}{m_8^2}\right) . \end{aligned}$$

We model the couplings $g_1$, $g_2$ such that they behave as $O(p^2)$ based on a scalar resonance chiral Lagrangian analogous to the one introduced in Ref. [48], which gives

42 $$\begin{aligned} g_1= & {} \frac{\sqrt{6}}{3F_\pi ^2}( c'_d\,(s-m_\eta ^2-m_\pi ^2)+2c'_m\,m_\pi ^2),\nonumber \\ g_2= & {} \frac{1}{F_\pi ^2}( c'_d\,(s-2m_K^2)+2c'_m\,m_K^2). \end{aligned}$$

We will discuss in Sect. 5 how the phenomenological parameters may be determined from experimental information on the properties of the $a_0(980)$, $a_0(1450)$ resonances as well as chiral constraints on the amplitudes and on the $I=1$ scalar form factor. Figure 3 illustrates how the unitary amplitudes parametrised as described above correctly match with the NLO chiral amplitudes at low energy.

## Phenomenological determination of the phase shifts and inelasticity and the $I=1$ scalar form factor

### Experimental information on $\pi \eta \rightarrow \pi \eta$ and $\pi \eta \rightarrow K{\bar{K}}$ scattering

Let us first consider the $\pi \eta \rightarrow \pi \eta$ amplitude below the $K{\bar{K}}$ threshold. In this region, $\eta \pi$ scattering should be approximately elastic. The $\pi \eta$ scattering phase shift below 1 GeV should be controlled by the values of the threshold parameters $a_0$, $b_0$ on the one hand and the properties of the $a_0(980)$ resonance on the other. We will consider that the values of $a_0$, $b_0$ corresponding to the set of $L_i$ with small $L_4$, $L_6$ (set (B); see Table 1) are the most plausible. In this case, $a_0$ and $b_0$ are both positive and one expects that the phase shift will be positive in the whole elastic region. A different possibility was investigated in Ref. [49].

The $a_0(980)$ is a well established resonance but its shape is not well described by a simple Breit–Wigner formula because of the vicinity of the $K{\bar{K}}$ threshold. This partly explains the dispersion in the values of the mass and width quoted by the PDG [50]: $m_{a_0}=980\pm 20$ MeV, $\varGamma _{a_0}=[50$–100] MeV. A comparison of a number of determinations of the $T_{11}$ amplitude near the $K{\bar{K}}$ threshold based, in particular, on the popular Flatté model [51] is performed in Ref. [52]. The corresponding $\eta \pi$ phase shifts are plotted on Fig. 10 of that reference, from which one can deduce that the value of the phase shift at the $K{\bar{K}}$ threshold lies around 90$^{\circ }$,

43 $$\begin{aligned} \delta _{11}(2m_K)=(90\pm 20)^{\circ }. \end{aligned}$$

This is also so in the models of Refs. [8, 9] which give, respectively, $\delta _{11}(2m_K)=95^\circ$ and $\delta _{11}(2m_K)=77^\circ$.

The $a_0(980)$ resonance corresponds to poles of the amplitude in the complex plane on the second and on the third Riemann sheets which can both be near the physical region since the mass is very close to the $K{\bar{K}}$ threshold. For definiteness, we will rely here on the recent determination by the KLOE collaboration [53]. It is based on measurements of the $\phi \rightarrow \eta \pi \gamma$ decay amplitude with both high precision and high statistics. Based on the best fit performed in Ref. [53] (using the theoretical model from Ref. [54]) the location of the poles can be deduced to be

44 $$\begin{aligned} \sqrt{s^{\mathrm{II}}_{a_0(980)}}= & {} (994\pm 2 -i\,(25.4\pm 5.0))~\hbox {MeV},\nonumber \\ \sqrt{s^{\mathrm{III}}_{a_0(980)}}= & {} (958\pm 13 -i\,(60.8\pm 11.5))~\hbox {MeV}. \end{aligned}$$

In the [1–2] GeV-energy region, a second resonance, the $a_0(1450)$, first reported in Ref. [55], was later identified in $\bar{p}p$ decays at rest (e.g. [56–58]; see also [59] who re-analysed the data). This resonance should correspond to a pole on the third Riemann sheet. Based on the value of the mass and width quoted in the PDG, we can estimate

45 $$\begin{aligned} \sqrt{s_{a_0(1450)}^{\mathrm{III}}}=(1474\pm 19 -i\,(133\pm 7))~\hbox {MeV}. \end{aligned}$$

A further property of the $a_0(1450)$ is that it has approximately equal decay widths into $\pi \eta$ and into $K{\bar{K}}$. We will implement this feature by requiring that the $J=0$ cross sections for $\eta \pi \rightarrow \eta \pi$ and $\eta \pi \rightarrow K{\bar{K}}$ should be approximately equal when $\sqrt{s}=1.474$ GeV. In our two-channel framework, these cross sections have the following expressions in terms of the phase shifts and the inelasticity parameter:

46 $$\begin{aligned}&\sigma (\eta \pi \rightarrow \eta \pi )=\frac{\pi }{p_{\eta \pi }^2} \vert \eta \,\hbox {e}^{2i\delta _{11}}-1\vert ^2,\nonumber \\&\sigma (\eta \pi \rightarrow K{\bar{K}})= \frac{\pi }{p_{\eta \pi }^2}(1-\eta ^2), \end{aligned}$$

and we expect that $\eta$ should reach a minimum at the mass of the $a_0(1450)$ resonance. If the minimum is close to zero, the two cross sections will be approximately equal.^4^ In this situation, we expect a rapid variation of the phase shifts $\delta _{11}$, $\delta _{22}$ (possibly becoming discontinuous if $\eta =0$) at the energy $\sqrt{s}=m_{a_0(1450)}$. In contrast, the sum of the two phase shifts (which is also the phase of $S_{12}$) should be a smoothly varying function. It is convenient to characterise the global behaviour of the *S*-matrix in the [1–2] GeV region in terms of the value of this phase sum $\delta _{11}+\delta _{12}$ when $\sqrt{s}=m_{a_0(1450)}$

47 $$\begin{aligned} \delta _{12}\equiv \left. \delta _{11}(\sqrt{s}) +\delta _{22}(\sqrt{s}) \right| _{\sqrt{s}=m_{a_0(1450)}}. \end{aligned}$$

Let us now return to the parametrisation of the *T*-matrix described in Sect. 4. The *T*-matrix elements in this model have analyticity properties and can be defined away from the physical region, in the complex energy plane. Using Eq. (35), the poles of the *T*-matrix correspond to the zeros of the determinant

48 $$\begin{aligned} \varDelta (s)=\hbox {det}[ 1-\varvec{K}(s)\varvec{\varPhi }(s)]. \end{aligned}$$

Recalling that the extensions of the loop functions ${\bar{J}}_{PQ}$ to the second Riemann sheet are defined as

49 $$\begin{aligned} {\bar{J}}_{PQ}^{\mathrm{II}}(s)= {\bar{J}}_{PQ}(s) + \frac{i\,\sqrt{\lambda _{PQ}(s)}}{8\pi \,s}, \end{aligned}$$

the extension of the *T*-matrix elements to the second Riemann sheet is performed by replacing ${\bar{J}}_{\eta \pi }(s)$ by ${\bar{J}}_{\eta \pi }^{\mathrm{II}}(s)$ in the matrix $\varvec{\varPhi }$. Similarly, the extension to the third Riemann sheet is performed by replacing both ${\bar{J}}_{\eta \pi }$ and ${\bar{J}}_{K{\bar{K}}}$ by ${\bar{J}}_{\eta \pi }^{\mathrm{II}}$ and ${\bar{J}}_{K{\bar{K}}}^{\mathrm{II}}$ in $\varvec{\varPhi }$.

This *T*-matrix model involves the phenomenological parameters: $\alpha _1$, $\alpha _2$, $\beta _1$, $\beta _2$, $m_8$, $c'_d$, $c'_m$. For simplicity, we will keep the ratio $c'_m/c'_d$ fixed and allow only six parameters to vary. We determine them by imposing six conditions on the *T*-matrix:

- As the first four conditions, we impose the requirement that the real and imaginary parts of the poles ${s^{\mathrm{II}}_{a_0(980)}}$ and ${s^{\mathrm{III}}_{a_0(1450)}}$ be reproduced.
- As a fifth condition, we impose the requirement that the minimum of the inelasticity parameter at $\sqrt{s}=m_{a_0(1450)}$ be close to zero (in practice, we used $\eta _{min}\approx 0.05$, as in Ref. [60]).
- As a final condition, we choose a value for the phase $\delta _{12}$ as defined in Eq. (47).

**Fig. 4:**
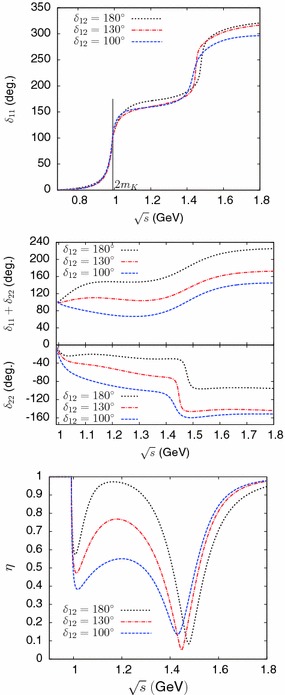
Phases $\delta _{11}$, $\delta _{22}$, their sum and the inelasticity $\eta$ from the *T*-matrix model of Sect. 4 corresponding to several imposed values of $\delta _{12}$ (defined in Eq. (47))

Within this model, having imposed the first five conditions, the value of $\delta _{12}$ is found to be bounded from above: $\delta _{12}\,{ \lesssim }\, 205^\circ$. In addition, consistently with our assumption that most of the phase variations should take place below 2 GeV, it seems plausible that the phase sum $\delta _{11}+\delta _{22}$ should not be smaller than its value at the mass of the $a_0(980)$, i.e., one should have $\delta _{12}\,{ \gtrsim }\, 90^\circ$. Figure 4 shows results from this model for the phases $\delta _{11}$, $\delta _{22}$ and the inelasticity $\eta$ as a function of energy, corresponding to several different imposed values of $\delta _{12}$. One observes that the two phases $\delta _{11}$, $\delta _{22}$ undergo a sharp variation, in opposite directions, close to the mass of the $a_0(1450)$ resonance. The figure illustrates a pattern where $\delta _{11}$ increases while $\delta _{22}$ decreases. However, a small modification of the phenomenological parameters which enter into the *T*-matrix model can lead to a pattern with a reversed behaviour (with $\delta _{11}$ decreasing and $\delta _{22}$ increasing), which would then be similar to the one obtained in Ref. [60]. In contrast, the phase sum, $\delta _{11}+\delta _{22}$, is completely stable and always increases smoothly as an effect of the resonance. This ambiguity, which can be viewed as a $\pm \pi$ ambiguity in the individual definition of $\delta _{11}$ and $\delta _{22}$, does also not affect observables, in particular, the determination of the form factors.

**Table 2:** Parameters of the *T*-matrix model corresponding to five fixed conditions (see text) and several input values of the phase $\delta _{12}$. The parameters $c'_m$, $c'_d$ are given in terms of $\lambda$ by $c'_d= \lambda c_d^0$, $c'_m= \lambda c_d^0/2$ with $c_d^0= 28$ MeV

$\delta _{12}$	$\alpha _1$	$\alpha _2$	$\beta _1~(\hbox {GeV}^{-1})$	$\beta _2~(\hbox {GeV}^{-1})$	$m_8~(\hbox {GeV})$	$\lambda$
200$^\circ$	0.6265	0.0988	0.2495	0.1476	1.0571	0.5704
175$^\circ$	0.7427	0.0781	0.3085	$-$0.0590	1.0913	0.8176
150$^\circ$	0.8444	0.0467	0.2773	$-$0.2085	1.1258	1.1017
125$^\circ$	0.8765	0.0016	0.2134	$-$0.3606	1.1834	1.6856
100$^\circ$	1.0993	$-$0.5055	$-$0.0358	$-$0.2722	1.5130	5.7024

Numerical values for the set of six parameters $\alpha _i$, $\beta _i$, $m_8$, $c'_d$ corresponding to several input values of $\delta _{12}$ in the range $90^\circ \le \delta _{12}\le 205^\circ$ are given in Table 2. The *T*-matrix is not very sensitive to the value of the parameter $c'_m$. Very similar results are obtained if one sets $c'_m=0$ or $c'_m=c'_d$. The numerical results shown in the table correspond to taking $c'_m=c'_d/2$. In this model, the pole of the *K*-matrix corresponds to two physical resonances. Table 2 shows that the mass parameter of the pole, $m_8$, varies between 1 and 1.5 GeV, while the value of the parameter $c'_d$ varies in a rather large range from 16 to 160 MeV, depending on the input value of the phase $\delta _{12}$.

**Fig. 5:**
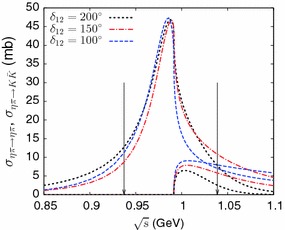
Cross sections for $\eta \pi \rightarrow \eta \pi$ and $\eta \pi \rightarrow K{\bar{K}}$ in the vicinity of the $a_0(980)$ resonance from the *T*-matrix model, depending on the input value of $\delta _{12}$. *The arrows* show the integration limits used to define the branching fraction (50)

The properties of the $a_0(980)$ resonance (apart from the pole position on the second Riemann sheet which is held fixed) depend on the value of $\delta _{12}$. Figure 5 shows the two cross sections $\sigma _{\eta \pi \rightarrow \eta \pi }$, $\sigma _{\eta \pi \rightarrow K{\bar{K}}}$ in the vicinity of the $a_0(980)$ resonance peak. We estimate the branching fraction $B_{K{\bar{K}}/\eta \pi }= \varGamma _{a_0\rightarrow K{\bar{K}}}/\varGamma _{a_0\rightarrow \eta \pi }$ in a simple way in terms of integrals over these cross sections:

50 $$\begin{aligned} B_{K{\bar{K}}/\eta \pi }=\dfrac{\int _{E^-}^{E^+} \sigma _{\eta \pi \rightarrow K{\bar{K}}}(E) \,\mathrm{d}E}{\int _{E^-}^{E^+} \sigma _{\eta \pi \rightarrow \eta \pi }(E) \,\mathrm{d}E} \end{aligned}$$

with $E^\pm = m_{a_0}\pm \varGamma _{a_0}$. In this formula, we set $m_{a_0}=988$ MeV, which corresponds to the resonance peak in the cross sections and $\varGamma _{a_0}=50.8$ MeV corresponding to twice the imaginary part of the pole position. We collect in Table 3 the results for the branching fraction corresponding to different input values of $\delta _{12}$. The agreement with the experimental average quoted in the PDG, $B^{exp}_{K{\bar{K}}/\eta \pi }=0.183\pm 0.024$ is qualitatively reasonable, in particular for the smaller values of $\delta _{12}$. We also indicate in the table the positions of the $a_0(980)$ pole on the third Riemann sheet (recall that the pole position on the second Riemann sheet is fixed), which is seen to move away from the real axis as $\delta _{12}$ is decreased.

**Table 3:** Some properties of the $a_0(980)$: values of the $\eta \pi /K{\bar{K}}$ branching fraction and position of the pole on the third Riemann sheet depending on the input value of the phase $\delta _{12}$

$\delta _{12}$	$B_{\eta \pi /K{\bar{K}}}$	$\sqrt{s^{\mathrm{III}}_{a_0}}~(\hbox {MeV})$
200$^\circ$	0.095	$1022-i\,62$
175$^\circ$	0.127	$1020-i\,93$
150$^\circ$	0.148	$1009-i\,129$
125$^\circ$	0.170	$972 -i\,192$
100$^\circ$	0.187	$749 -i\,376$

### Scalar form factors and the $\eta \pi$ scalar radius

In order to solve the integral equations (30) we must also define $\delta _{11}(s)$, $\delta _{22}(s)$, $\eta (s)$ for energies above the mass of the $a_0(1450)$ resonance such that the asymptotic conditions (32) are satisfied. For this purpose, we define a mapping *u*(*s*) such that $0\le u \le 1$ when $s_1 \le s \le \infty$ and then perform simple polynomial interpolations of the functions $\delta _{11}$, $\delta _{22}$, $\eta$ in terms of the variable *u* (see Appendix C for more details, in practice we used $\sqrt{s_1}=1.8$ GeV). For a given value of the phase $\delta _{12}$, the *T*-matrix is completely specified and one can derive the two scalar form factors by solving Eq. (30).

**Fig. 6:**
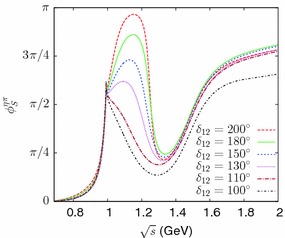
Phase of the form factor $F_S^{\eta \pi }$ obtained from solving the integral equations (30) with several input values of the phase $\delta _{12}$ (see Eq. (47)) in the *T*-matrix

The form factors turn out to be rather sensitive to the value of $\delta _{12}$. Figure 6 illustrates the numerical results for the phase of the $\eta \pi$ scalar form factor, $\phi _S^{\eta \pi }$, corresponding to different input values of $\delta _{12}$. The phase of the form factor displays a dip located in between the two $a_0$ resonances. This behaviour is qualitatively similar to the one observed for the scalar form factor phases in the cases of the $\pi \pi$ or $K\pi$. A detailed discussion can be found in Ref. [61]. The phase $\phi _S^{\eta \pi }$ displays a bump, before the dip, which disappears when the input value of $\delta _{12}$ is smaller than ${\simeq }130^\circ$. Given the phase integral representation (29), we expect the $\eta \pi$ scalar radius to decrease when $\delta _{12}$ decreases. Numerical values of the scalar radii for the $\eta \pi$ and the $K{\bar{K}}$ form factors are displayed in Table 4 for given values of $\delta _{12}$ in the range [100$^\circ$–200$^\circ$]. In all cases, the dispersive result for ${\langle r^2 \rangle }^{\eta \pi }_S$ exceeds the $O(p^4)$ chiral value (8) (the same also holds for the $K{\bar{K}}$ scalar radius). However, one must also take into account the chiral corrections of order $p^6$ (or higher), the typical size of which can be as large as 20–$30~\%$. In the dispersive evaluation, even if the *T*-matrix elements were known exactly below 2 GeV, an error would arise from the asymptotic region. This is easily seen from the phase integral expression (29). The contribution to the $\eta \pi$ scalar radius from the integration region $\sqrt{s'}>2$ GeV is relatively large ${\simeq } 30~\%$ and this could generate an overall uncertainty for ${\langle r^2 \rangle }_S^{\eta \pi }$ of the order of $15~\%$. The conclusion, then, is that the chiral result and the dispersive evaluation can be perfectly compatible provided the phase $\delta _{12}$ lies in the following restricted range: $90^\circ \,{ \lesssim }\, \delta _{12} \,{ \lesssim }\, 125^\circ$.

**Table 4:** Results for the scalar radii obtained from solving Eq. (30) for the form factors depending on the input value for the phase $\delta _{12}$

$\delta _{12}$	200$^\circ$	175$^\circ$	150$^\circ$	125$^\circ$	100$^\circ$
${\langle r^2 \rangle }^{\eta \pi }_S\,(\hbox {fm}^2)$	0.185	0.176	0.166	0.150	0.122
${\langle r^2 \rangle }^{K{\bar{K}}}_S\,(\hbox {fm}^2)$	0.253	0.248	0.245	0.233	0.209

**Fig. 7:**
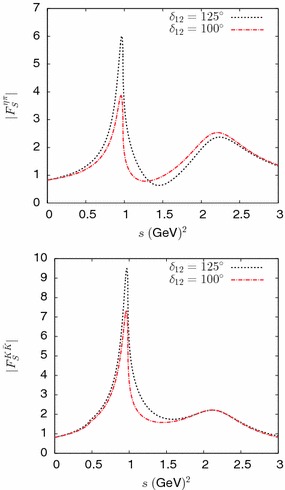
Absolute values of the form factors $F_S^{\eta \pi }$ (*top*) and $F_S^{K{\bar{K}}}$ (*bottom*) computed from our *T*-matrix model, corresponding to two input values of the phase $\delta _{12}$

Finally, Fig. 7 shows the absolute values of the form factors $F_S^{\eta \pi }$, $F_S^{K{\bar{K}}}$. The size of the peak associated with $a_0(980)$ resonance is seen to be sensitive to value of the phase $\delta _{12}$. We have verified that the associated spectral function agrees with the one given in Ref. [31] in the energy range $s < 1.5~\hbox {GeV}^2$ when $\delta _{12}\simeq 100^\circ$.

## Conclusions

We have proposed a model for the $\eta \pi$ scattering *T*-matrix in the *S*-wave which satisfies elastic unitarity below the $K{\bar{K}}$ threshold and two-channel unitarity above. The model is constrained by experimental inputs on the properties of the two resonances $a_0(980)$, $a_0(1450)$ and by chiral symmetry at low energy. In the simple *K*-matrix type framework which we have adopted it is possible to match correctly the two amplitudes $\eta \pi \rightarrow \eta \pi$, $\eta \pi \rightarrow K{\bar{K}}$ with the chiral expansion at NLO while in the case of $K{\bar{K}}\rightarrow K{\bar{K}}$, the matching is only approximate (see Sect. 4). Such a *T*-matrix could be realistic in an energy range $\sqrt{s}\,{ \lesssim }\, 1.3$ GeV, where the inelasticity is effectively dominated by the $K{\bar{K}}$ channel. Formally, however, it is convenient to extend the model up to infinite energies such as to allow for a minimal solution of the associated Muskhelishvili–Omnès problem.

A specific prediction of three-flavour ChPT is that the $$J=0$$$$\eta \pi \rightarrow \eta \pi $$ scattering length is very small, while the scattering range vanishes at leading order. The detailed predictions for these quantities at NLO are very sensitive to the values of the couplings $$L_4$$, $$L_6$$, which are $$1/N_c$$ suppressed. We have used here the values of $$L_4$$, $$L_6$$ which are favoured by lattice QCD simulations. It would be a particularly interesting test of the chiral expansion, obviously, to have a verification of the $$\eta \pi $$ scattering length also from lattice QCD.

A supplementary chiral constraint which we have used is associated with the $\eta \pi$ scalar isovector form factor. We have computed this scalar form factor from our two-channel *T*-matrix by solving the relevant Muskhelishvili–Omnès integral equations. While this model ignores other relevant inelastic channels (like $\pi \eta '$), it is nevertheless plausible that it should be able to describe how the phase of the form factor behaves in approximately the same energy range where the *T*-matrix is realistic. Above this point, the model simply serves to interpolate the form factor phase monotonically towards its known asymptotic value. We find that the small value of the $\eta \pi$ scalar radius in ChPT at NLO can be understood in this approach and that this requirement constrains the increase of the sum of *S*-matrix phases $\delta _{11}+\delta _{22}$ in the 1–2-GeV energy region. One should keep in mind the uncertainties on the size of the NNLO effects on the ChPT side and those from the energy range above 2 GeV on the dispersive side. The dispersive calculation suggest that the NNLO corrections to ${\langle r^2 \rangle }_S^{\eta \pi }$ should tend to increase its size. It would again be extremely useful to have results from lattice QCD for this quantity.

The computation of the $2\times 2$ Omnès matrix $\varvec{\varOmega }$ is a straightforward extension of the form factor calculation. In principle, the $\varvec{\varOmega }$ matrix allows one to treat the $\eta \pi$ rescattering effects in a unified way, in a number of processes for which recent measurements have been performed like $\eta '\rightarrow \eta \pi \pi$, $\phi \rightarrow \eta \pi \gamma$ or $\gamma \gamma \rightarrow \eta \pi$. The consideration of $\eta \pi$ rescattering is also necessary in the case of the $\eta \rightarrow 3\pi$ amplitude in order to account for $a_0$–$f_0$ mixing within a dispersive approach. The $\eta \pi$ scalar form factor itself appears in the isospin suppressed $\tau \rightarrow \eta \pi \nu$ amplitude, along with an electromagnetic induced scalar form factor (and the vector form factor). This decay mode has not yet been observed but could possibly be studied at the super-B or future charm-tau factory.

## Appendix A: The $I=1$ scalar form factors at NLO

We consider the two form factors defined in Eq. (2). At leading order of the chiral expansion, the form factors are simply constant,

A.1 $$\begin{aligned} F_S^{\eta \pi }(0)=\frac{\sqrt{6}}{3},\quad F_S^{K{\bar{K}}}(0)=1\qquad (LO). \end{aligned}$$

Computing and adding the next-to-leading order corrections, the form factors can be written as

A.2 $$\begin{aligned} F_S^{\eta \pi }(s)= & {} F_S^{\eta \pi }(0)\, \left\{ 1 + {s\over F_\pi ^2} \, \left[ 4\,L^r_{5} - \frac{3}{4}\,\frac{1}{16\pi ^2}(1+ L_K)\right] \right. \nonumber \\&\left. -\frac{1}{4F_\pi ^2}\,(4\,m_K^2-3\,s)\,{\bar{J}}_{KK}(s) +\frac{m_\pi ^2}{3F_\pi ^2}\,{\bar{J}}_{\pi \eta }(s)\right\} ,\nonumber \\ \end{aligned}$$

A.3 $$\begin{aligned} F_S^{K{\bar{K}}}(s)= & {} F_S^{K{\bar{K}}}(0)\,\left\{ 1 + \frac{s}{F_\pi ^2} \, \left[ 4\,L^r_{5} -\frac{1}{4} \frac{1}{16\pi ^2}(1\right. \right. \nonumber \\&\left. +2 \,R_{\pi \eta }+2\,L_\eta + L_K)\right] + \frac{s}{4F_\pi ^2}\, {\bar{J}}_{KK}(s)\, \nonumber \\&\left. - \frac{1}{6F_\pi ^2}\,(4\,m_K^2 - 3\,s){\bar{J}}_{\pi \eta }(s) \right\} , \end{aligned}$$

where we have introduced the notation

A.4 $$\begin{aligned} L_P=\log \frac{m_P^2}{\mu ^2},\ R_{PQ}=\frac{m_P^2\,\log (m_P^2/m_Q^2)}{m_P^2-m_Q^2}, \end{aligned}$$

and ${\bar{J}}_{PQ}(s)$ are the loop functions defined to vanish at $s=0$ (we use the same notation as Ref. [23]),

A.5 $$\begin{aligned} {\bar{J}}_{PQ}(s)= & {} \frac{s}{16\pi ^2}\int _{(m_P+m_Q)^2}^\infty \mathrm{d}s'\, \frac{\sqrt{\lambda _{PQ}(s')}}{(s')^2(s'-s)}\nonumber \\= & {} \frac{1}{16\pi ^2}\left[ 1+\left( \frac{\varSigma _{PQ}}{\varDelta _{PQ}} -\frac{\varDelta _{PQ}}{s}\right) \log \frac{m_P}{m_Q}\right. \nonumber \\&\left. +\frac{\sqrt{\lambda _{PQ}(s)}}{2s}\,\log \frac{\varSigma _{PQ}-s+\sqrt{\lambda _{PQ}(s)}}{\varSigma _{PQ}-s-\sqrt{\lambda _{PQ}(s)}}\right] \end{aligned}$$

with

A.6 $$\begin{aligned}&\varSigma _{PQ}=m_P^2+m_Q^2,\quad \varDelta _{PQ}=m_P^2-m_Q^2,\nonumber \\&\lambda _{PQ}(s)=s^2-2\varSigma _{PQ}\,s+\varDelta _{PQ}^2. \end{aligned}$$

The expression for $F_S^{\eta \pi }(0)$ is given by

A.7 $$\begin{aligned}&F_S^{\eta \pi }(0)={\sqrt{6}\over 3}\bigg \{ 1 + {m_K^2\over F_\pi ^2} \, \left[ - 64\,L^r_{7}+ 32\,L^r_{6}\right. \nonumber \\&\quad \left. -\frac{32}{3}\,L^r_{5} - 16\,L^r_{4} + \frac{1}{16\pi ^2}\,\left( 1 - \frac{2}{9}\,L_\eta + 2\,L_K\right) \right] \nonumber \\&\quad + {m_\pi ^2\over F_\pi ^2} \, \left[ 32\,L^r_{8} + 64\,L^r_{7} + 16\,L^r_{6} - \frac{16}{3}\,L^r_{5}- 8\,L^r_{4}\right. \nonumber \\&\quad \left. \left. +\frac{1}{16\pi ^2}\,\left( -\frac{1}{3}\,R_{\pi \eta } - \frac{5}{18}\,L_\eta - \frac{1}{2}\,L_\pi \right) \right] \right\} \end{aligned}$$

and the expression of $F_S^{K{\bar{K}}}(0)$ reads

A.8 $$\begin{aligned}&F_S^{K{\bar{K}}}(0)= 1+ \frac{m_K^2}{F_\pi ^2} \, \left[ 16\,(2\,L^r_{8}-L^r_{5}) + 16\,(2\,L^r_{6}-L^r_{4})\right. \nonumber \\&\quad \left. + \frac{1}{16\pi ^2}\left( \frac{2}{3}\,R_{\pi \eta } + \frac{10}{9}\,L_\eta \right) \right] + \frac{m_\pi ^2}{F_\pi ^2} \, \left[ 8\,(2\,L^r_{6}-L^r_{4})\right. \nonumber \\&\quad \left. +\frac{1}{16\pi ^2}\left( -\frac{1}{9}\,L_\eta \right) \right] . \end{aligned}$$

### Appendix A.1 Remarks on $F_S^{K{\bar{K}}}(0)$, $F_S^{\eta \pi }(0)$

The value of $F_S^{K{\bar{K}}}(0)$ can be simply related to the $K^0-K^+$ mass difference. Indeed, using isospin symmetry, one can express the form factor $F_S^{K{\bar{K}}}$ as

A.9 $$\begin{aligned} B_0 F_S^{K{\bar{K}}}(s)=\left\langle {\frac{{K^0}{{\bar{K}}^0}-{K^+}{K^-}}{\sqrt{2}}\left| \frac{{\bar{u}}{u}-\bar{d}{d}}{\sqrt{2}}\right| 0}\right\rangle . \end{aligned}$$

Then, writing the quark masses as

A.10 $$\begin{aligned} m_u= \hat{m}-\frac{1}{2}\varDelta _{du},\quad m_d= \hat{m}+\frac{1}{2}\varDelta _{du}, \end{aligned}$$

Feynman–Hellmann’s theorem yields the following relation:

A.11 $$\begin{aligned} B_0 F^{K{\bar{K}}}_S(0)=\frac{\mathrm{d}}{\mathrm{d}\varDelta _{du}}\left( M^2_{K^0}-M^2_{K^+}\right) . \end{aligned}$$

One can easily reproduce Eq. (A.8) using this relation and the chiral formula for the mass difference $M^2_{K^0}-M^2_{K^+}$ from Ref. [23]. Using this formula, one can also derive an alternative expression for $F_S^{K{\bar{K}}}(0)$,

A.12 $$\begin{aligned} F_S^{K{\bar{K}}}(0)= \frac{(m_K^2-m_\pi ^2)}{(m_s-\hat{m})B_0}\times \frac{r_2+1}{r+1} \end{aligned}$$

where *r* is the quark mass ratio $m_s/\hat{m}$ and $r_2= 2m_K^2/m_\pi ^2-1$ is the value of this ratio at chiral order $p^2$. The deviation of the value of $F_S^{K{\bar{K}}}(0)$ from 1 can thus be interpreted as a measure of the size of the $O(p^4)$ corrections in the chiral expansion of the mass difference $m_K^2-m_\pi ^2$. Table 5 below shows that, if one uses the set of $L_i$ with large $L_4$, $L_6$, this correction is rather large (of the order of 40 %).

We can also perform a verification of the value of $F_S^{\eta \pi }(0)$. Using the Ward identity in pure QCD,

A.13 $$\begin{aligned} i\partial _\mu {\bar{u}}\gamma ^\mu {d}=(m_d-m_u)\,{\bar{u}}{d}, \end{aligned}$$

we can relate $F^{\eta \pi }_S(0)$ to the value at zero of the $\eta \pi$ vector form factor $f_+^{\eta \pi }$ (normalised as in Ref. [62]) when $e^2=0$

A.14 $$\begin{aligned} F_S^{\eta \pi }(0)= \frac{\sqrt{2}(m_\eta ^2-m_\pi ^2)}{(m_d-m_u)B_0} \left. f_+^{\eta \pi }(0)\right| _{e^2=0}. \end{aligned}$$

Inserting the chiral expansion expressions for $m_\eta ^2$, $m_\pi ^2$ from Ref. [23] and $f_+^{\eta \pi }(0)$ from Ref. [62] one can recover Eq. (A.7).

The numerical values of $F_S^{\eta \pi }(0)$, $F_S^{K{\bar{K}}}(0)$ are needed as input in order to solve the integral equations (30) for the scalar form factors. The values at $s=0$ are rather sensitive to the $1/N_c$ suppressed couplings $L_4$, $L_6$ as can be seen from Table 5 below. However, the determination of the scalar radii ${\langle r^2 \rangle }_S^{\eta \pi }$, ${\langle r^2 \rangle }_S^{K{\bar{K}}}$ from the integral equations depends only on the ratio $F_S^{\eta \pi }(0)/F_S^{K{\bar{K}}}(0)$. It is easy to verify that this ratio is independent from $L_4$, $L_6$ at NLO.

**Table 5:** Numerical values of $F_S^{\eta \pi }(0)$, $F_S^{K{\bar{K}}}(0)$ in the chiral expansion at LO and at NLO using two sets of low-energy couplings (see Table 1)

	$O(p^2)$	Small $L_4^r$, $L_6^r$	Large $L_4^r$, $L_6^r$
$F_S^{\eta \pi }(0)$	0.816	0.826	1.421
$F_S^{K{\bar{K}}}(0)$	1	0.816	1.428

### Appendix A.2: Expression of $\delta _2$

We reproduce here the detailed expression (as given in eq. 6.2 of Ref. [24]) for the term $\delta _2$, which appears in the chiral expansion of the $K\pi$ scalar radius at order $p^4$ (see Eq. (7)),

A.15 $$\begin{aligned} \delta _2= & {} \frac{-1}{192\pi ^2F_\pi ^2}\left[ 15\,h_2\left( \frac{m_\pi ^2}{m_K^2}\right) +\frac{19\,m_K^2+3\,m_\eta ^2}{m_K^2+m_\eta ^2}\right. \nonumber \\&\quad \left. \times h_2\left( \frac{m_\eta ^2}{m_K^2}\right) -18\right] , \end{aligned}$$

with

A.16 $$\begin{aligned} h_2(x)=\frac{3}{2}\left( \frac{1+x}{1-x}\right) ^2 +\frac{3x\,(1+x)}{(1-x)^3}\log (x). \end{aligned}$$

## Appendix B: NLO contributions to $I=1$ scattering amplitudes

We give below the expressions of the chiral NLO contributions to the one-variable functions associated with the amplitudes $\eta \pi ^+\rightarrow \eta \pi ^+$, $\eta \pi ^+\rightarrow {\bar{K}}^0 K^+$ and ${\bar{K}}^0 K^+\rightarrow {\bar{K}}^0 K^+$.

### Appendix B.1: The $\eta \pi ^+\rightarrow \eta \pi ^+$ amplitude

The $O(p^4)$ part of the amplitude was written in terms of the two functions $U_0^{11}$, $W_0^{11}$ (Eq. (11)). They can be expressed as follows:

B.1 $$\begin{aligned} U_0^{11}(s)= & {} {\frac{1}{F_\pi ^4}}\left\{ (s-\varSigma _{\eta \pi })^2 \, \left[ 4\,(L^r_{2}+L^r_{3}/3)\right. \right. \nonumber \\&\left. - \frac{3}{8} \frac{1}{16\pi ^2}\,( 1+L_K)\right] + \frac{1}{9}{\bar{J}}_{\pi \eta }(s)\,m_\pi ^4\nonumber \\&\left. + \frac{1}{24}\,{\bar{J}}_{KK}(s) \, (4\,m_K^2-3\,s)^2\right\} , \end{aligned}$$

and

B.2 $$\begin{aligned}&W_0^{11}(t)={\frac{1}{F_\pi ^4}}\left\{ (t-2\,m_\pi ^2)\,(t-2\,m_{\eta }^2) \, \left[ 4\,(2\,L^r_{1}+L^r_{3}/3)\right. \right. \nonumber \\&\quad \left. - \frac{3}{8} \frac{1}{16\pi ^2}\,( 1+ L_K)\right] + m_\pi ^2\,m_{\eta }^2 \, \left[ 32\left( -\,L^r_{7}+ \,L^r_{6}\right. \right. \nonumber \\&\quad \left. \left. - \frac{1}{6}\,L^r_{5} - \,L^r_{4}\right) + \frac{1}{16\pi ^2}\,\left( \frac{23}{18} + 2\,L_K- \frac{2}{9}\,L_\eta \right) \right] \nonumber \\&\quad + m_\pi ^4 \, \left[ 16\,L^r_{8} + 32\,L^r_{7} + \frac{1}{16\pi ^2}\,\left( -\frac{1}{9} - \frac{2}{9}\,R_{\pi \eta }\right. \right. \nonumber \\&\quad \left. \left. -\frac{1}{6}\,L_K- \frac{1}{6}\,L_\eta - \frac{1}{2}\,L_\pi \right) \right] + t\,\varSigma _{\eta \pi } \, \left[ 8\,L^r_{4}\right. \nonumber \\&\quad \left. - \frac{1}{2} \frac{1}{16\pi ^2}\,( 1+L_K)\right] + t\,m_\pi ^2 \, \left[ \frac{1}{3}\,\frac{1}{16\pi ^2}\,\log \left( \frac{m_K^2}{m_\pi ^2}\right) \right] \nonumber \\&\quad - \frac{1}{6}{\bar{J}}_{\pi \pi }(t) \, \,m_\pi ^2\,(m_\pi ^2 - 2\,t) + \frac{1}{54} {\bar{J}}_{\eta \eta }(t) \, \,m_\pi ^2\,(16\,m_K^2 \nonumber \\&\quad \left. - 7\,m_\pi ^2) - \frac{1}{24} {\bar{J}}_{KK}(t) \, \,t\,(8\,m_K^2 - 9\,t)\right\} . \end{aligned}$$

### Appendix B.2: The $\eta \pi ^+ \rightarrow {{\bar{K}}^0}{K^+}$ amplitude

The three functions involved in the NLO contributions to the amplitude were denoted as $U_0^{12}$, $W_0^{12}$ and $W_1$. They can be expressed as

B.3 $$\begin{aligned}&U_0^{12}(s) = {-\frac{\sqrt{6}}{F_\pi ^4}}\left\{ + m_K^4 \,\left[ \frac{16}{3} \,L^r_{8} + \frac{32}{3} \,L^r_{7} - \frac{8}{9} \,L^r_{5} - \frac{2}{9} \,L^r_{3}\right. \right. \nonumber \\&\quad \left. + \frac{1}{16\pi ^2}\,\left( \frac{1}{72} + \frac{1}{2} \,L_\eta -\frac{11}{18} \,L_K\right) \right] + m_\pi ^2 \,m_K^2 \,\left[ - \frac{16}{3} \,L^r_{8}\right. \nonumber \\&\quad - \frac{32}{3} \,L^r_{7} + \frac{32}{9} \,L^r_{5} + \frac{4}{9} \,L^r_{3} + \frac{1}{16\pi ^2}\,\left( - \frac{1}{36} - \frac{5}{8} \,L_\eta + \frac{17}{72} \,L_\pi \right. \nonumber \\&\quad \left. \left. + \frac{1}{2} \,R_{\eta K} - \frac{13}{9}\,R_{\pi K}\right) \right] + m_\pi ^4 \,\left[ - \frac{2}{9} \,L^r_{3} + \frac{1}{16\pi ^2}\,\left( \frac{1}{72}\right. \right. \nonumber \\&\quad \left. \left. + \frac{1}{8} \,L_\eta +\frac{4}{9} \,L_K- \frac{41}{72} \,L_\pi - \frac{1}{6} \,R_{\eta K} + \frac{11}{18} \,R_{\pi K}\right) \right] \nonumber \\&\quad + s \,m_K^2 \,\left[ + \frac{1}{16\pi ^2}\,\left( - \frac{1}{12} - \frac{1}{8} \,L_\eta +\frac{1}{8} \,L_K- \frac{29}{96} \,R_{\eta K} \right. \right. \nonumber \\&\quad \left. \left. + \frac{67}{96}\,R_{\pi K}\right) \right] + s \,m_\pi ^2 \,\left[ - 2 \,L^r_{5} + \frac{1}{16\pi ^2}\,\left( \frac{1}{32} \,L_\eta + \frac{1}{16}\,L_K\right. \right. \nonumber \\&\quad \left. + \frac{9}{32} \,L_\pi - \frac{7}{96} \,R_{\eta K} + \frac{13}{96} \,R_{\pi K})\right] + s^2 \,\left[ + \frac{1}{16\pi ^2}\,\left( \frac{1}{16}\right. \right. \nonumber \\&\quad \left. \left. + \frac{3}{32} \,R_{\eta K} - \frac{5}{32} \,R_{\pi K}\right) \right] +(4 \,m_K^2-3 \,s)\,\left[ \frac{1}{36}\,{\bar{J}}_{\pi \eta }(s) \, \,m_\pi ^2\right. \nonumber \\&\quad \left. \left. + \frac{1}{48} \,{\bar{J}}_{KK}(s) \,s\right] \right\} \end{aligned}$$

and

B.4 $$\begin{aligned}&W_0^{12}(t)= {-\frac{\sqrt{6}}{F_\pi ^4}}\left\{ {\bar{J}}_{K\pi }(t) \, \left[ + \frac{1}{12} \,m_\pi ^4 - \frac{1}{6} \,t \,m_\pi ^2 + \frac{5}{64} \,t^2 \right. \right. \nonumber \\&\quad + \frac{1}{12} \,\varDelta _{K\pi } \,m_\pi ^2 - \frac{1}{12} \,\varDelta _{K\pi } \,t + \frac{1}{16} \,\frac{\varDelta _{K\pi }^2}{t } \,m_\pi ^2- \frac{3}{64} \,\varDelta _{K\pi }^2\nonumber \\&\quad \left. + \frac{1}{32} \,\frac{\varDelta _{K\pi }^3}{t } + \frac{1}{48} \,\frac{\varDelta _{K\pi }^4}{t^2}\right] + {\bar{J}}_{K\eta }(t) \, \left[ - \frac{5}{36} \,m_\pi ^4 \right. \nonumber \\&\quad + \frac{1}{6} \,t \,m_\pi ^2 - \frac{3}{64} \,t^2 - \frac{1}{4} \,\varDelta _{K\pi } \,m_\pi ^2 + \frac{1}{6} \,\varDelta _{K\pi } \,t \nonumber \\&\quad \left. - \frac{7}{144} \,\frac{\varDelta _{K\pi }^2}{t } \,m_\pi ^2 - \frac{43}{576} \,\varDelta _{K\pi }^2 - \frac{19}{288} \,\frac{\varDelta _{K\pi }^3}{t } + \frac{1}{432} \,\frac{\varDelta _{K\pi }^4}{t^2}\right] \nonumber \\&\quad \left. - \frac{1}{48} \,{\bar{J}}'_{K\pi }(0) \,\frac{\varDelta _{K\pi }^4}{t } - \frac{1}{432} \,{\bar{J}}'_{K\eta }(0) \,\frac{\varDelta _{K\pi }^4}{t } \right\} , \end{aligned}$$

and, finally,

B.5 $$\begin{aligned}&W_1(t)={-\frac{\sqrt{6}}{F_\pi ^4}}\left\{ \, t \, \left[ + \frac{1}{3} \,L^r_{3} + \frac{1}{16\pi ^2}\,\left( - \frac{1}{48} + \frac{1}{16} \,R_{\eta K}\right. \right. \right. \nonumber \\&\quad \left. \left. -\frac{1}{16} \,R_{\pi K}\right) \right] - \frac{1}{64} \, {\bar{J}}_{K\pi }(t) \, \frac{\lambda _{K\pi }(t)}{t} - \frac{1}{64}\, {\bar{J}}_{K\eta }(t) \, \,\frac{\lambda _{K\eta }(t)}{t}\nonumber \\&\quad \left. + \frac{1}{16} \,{\bar{J}}'_{K\pi }(0) \,\varDelta _{K\pi }^2 + \frac{1}{144} \,{\bar{J}}'_{K\eta }(0) \,\varDelta _{K\pi }^2\right\} . \end{aligned}$$

### Appendix B.3: The amplitude ${{\bar{K}}^0}{K^+}\rightarrow {{\bar{K}}^0}{K^+}$

The $O(p^4)$ contributions to this amplitude involve five functions: $U_0^{22}$, $U_1$, $V_0$, $V_1$ and $W_0^{22}$ (see Eq. (11)). $U_0^{22}$ can be expressed as

B.6 $$\begin{aligned}&U_0^{22}(s) = {1\over F_\pi ^4}\left\{ m_K^4 \left[ 16\,L^r_{8} + 32\,L^r_{6}- 8\,L^r_{5} + \frac{1}{16\pi ^2}\left( - \frac{53}{36}\right. \right. \right. \nonumber \\&\quad \left. \left. +\frac{41}{72}\,L_\eta - \frac{3}{4}\,L_K- \frac{3}{8}\,L_\pi + \frac{11}{12}\,R_{\pi \eta }\right) \right] + s\,m_K^2\,\left[ - 8\,L^r_{4}\right. \nonumber \\&\quad \left. + \frac{1}{16\pi ^2}\,\left( \frac{3}{4} - \frac{1}{16}\,L_\eta +\frac{3}{8}\,L_K+ \frac{3}{16}\,L_\pi -\frac{5}{8}\,R_{\pi \eta }\right) \right] \nonumber \\&\quad + s\,m_\pi ^2 \, \left[ + 2\,L^r_{5} + \frac{1}{16\pi ^2}\,\left( - \frac{1}{16}\,L_\eta - \frac{5}{16}\,L_\pi \right) \right] \nonumber \\&\quad + (s - 2\,m_K^2 )^2 \,\left[ 2\,L^r_{3} + 4\,L^r_{2} + \frac{1}{16\pi ^2}\,\left( - \frac{1}{24} - \frac{3}{8}\,L_\eta \right. \right. \nonumber \\&\quad \left. \left. +\frac{1}{48}\,L_K- \frac{1}{48}\,L_\pi - \frac{3}{8}\,R_{\pi \eta }\right) \right] \nonumber \\&\quad \left. +\frac{1}{24}\,{\bar{J}}_{\pi \eta }(s)\,(4\,m_K^2 - 3\,s)^2 + \frac{1}{16}\,{\bar{J}}_{KK}(s)\,s^2\right\} . \end{aligned}$$

The function $U_1(s)$ reads

B.7 $$\begin{aligned}&U_1(s)={1\over F_\pi ^4}\left\{ m_K^2 \,\left[ 8\,L^r_{4} + \frac{1}{16\pi ^2}\,\left( - 1 + \frac{1}{16}\,L_\eta - \frac{3}{8}\,L_K\right. \right. \right. \nonumber \\&\quad \left. \left. - \frac{3}{16}\,L_\pi +\frac{1}{8}\,R_{\pi \eta }\right) \right] + m_\pi ^2 \,\left[ 2\,L^r_{5}+ \frac{1}{16\pi ^2}\,\left( - \frac{1}{16}\,L_\eta \right. \right. \nonumber \\&\quad \left. \left. -\frac{5}{16}\,L_\pi \right) \right] - \frac{1}{24}\,{\bar{J}}_{\pi \pi }(s)\,(4\,m_\pi ^2 - s) \nonumber \\&\quad \left. -\frac{1}{48}\,{\bar{J}}_{KK}(s)\,(4\,m_K^2 - s)\right\} . \end{aligned}$$

Next, the functions $V_0(t)$, $V_1(t)$ read

B.8 $$\begin{aligned} V_0(t)= & {} {1\over F_\pi ^4}\left\{ ( t- 2\,m_K^2)^2 \,\left[ 2\,L^r_{3} + 8\,L^r_{1}+ \frac{1}{16\pi ^2}\,\left( - \frac{29}{48}\right. \right. \right. \nonumber \\&\left. \left. - \frac{3}{32}\,L_\eta - \frac{11}{48}\,L_K- \frac{5}{96}\,L_\pi + \frac{3}{16}\,R_{\pi \eta }\right) \right] \nonumber \\&+ \frac{3}{32}\,{\bar{J}}_{\pi \pi }(t)\,t^2 + \frac{1}{288}\,{\bar{J}}_{\eta \eta }(t)\,(8\,m_K^2 - 9\,t)^2 \nonumber \\&\left. -\frac{1}{48}\,{\bar{J}}_{\pi \eta }(t)\, (4\,m_K^2 - 3\,t)^2 + \frac{1}{4}\,{\bar{J}}_{KK}(t)\,t^2\right\} ,\nonumber \\\end{aligned}$$

B.9 $$\begin{aligned} V_1(t)= & {} {1\over F_\pi ^4}\left\{ \frac{1}{48}\,{\bar{J}}_{\pi \pi }(t)\,(4\,m_\pi ^2 - t)\right. \nonumber \\&\left. -\frac{1}{12}\,{\bar{J}}_{KK}(t)\,(4\,m_K^2 - t)\right\} . \end{aligned}$$

Finally, the function $W_0^{22}(u)$ reads

B.10 $$\begin{aligned} W_0^{22}(u)= & {} {1\over F_\pi ^4}\left\{ ( 2\,m_K^2 - u)^2 \,\left[ 4\,L^r_{2} + \frac{1}{16\pi ^2}\,\left( - \frac{7}{24}\right. \right. \right. \nonumber \\&\left. \left. - \frac{17}{48}\,L_K-\frac{1}{48}\,L_\pi \right) \right] + \left. \frac{1}{4}\,{\bar{J}}_{KK}(u)\,(2\,m_K^2 - u)^2\right\} .\nonumber \\ \end{aligned}$$

## Appendix C: Asymptotic interpolation

We describe here the simple ansatz which we used for interpolating the *S*-matrix phases $\delta _{11}$, $\delta _{22}$ and the inelasticity $\eta$ in the asymptotic region $s_1 \le s < \infty$. Let *F*(*s*) be one of the functions $\delta _{11}(s)$, $\delta _{22}(s)$ or $\arccos (\eta (s))$. We introduce a point $s_2=s_1+\epsilon$ close to $s_1$ and we assume that $F(s_1)$, $F(s_2)$ are given. We denote $s_3=\infty$ and we use the asymptotic conditions (see Eq. (32))

C.1 $$\begin{aligned} \delta _{11}(s_3)= 2\pi ,\quad \delta _{22}(s_3)=0,\quad \eta (s_3)=1. \end{aligned}$$

Thus $F(s_3)$ is also known. We introduce a function *u*(*s*),

C.2 $$\begin{aligned} u(s)=\dfrac{1}{1+\log \dfrac{s}{s_1}}, \end{aligned}$$

which maps the range $[s_1,\infty ]$ onto the finite range [0, 1] and define *F*(*s*) through a simple Lagrange polynomial interpolation, i.e.,

C.3 $$\begin{aligned} F(s)=\sum _{i=1}^3 F(s_i) \frac{(u(s)-u_j)(u(s)-u_k)}{(u_i-u_j)(u_i-u_k)} \end{aligned}$$

with $u_i\equiv u(s_i)$ and *i*, *j*, *k* is a cyclic permutation of 1, 2, 3.

## Footnotes

1 This effect was first discussed in Ref. [4]. It can be seen as a superposition of the two physical resonances $a_0(980)$, $f_0(980)$ in the $\eta \pi \rightarrow \pi \pi$ scattering amplitude.

2 The following input numerical values are used throughout this paper (all in GeV): $m_\pi =0.139568$, $m_K=0.4957$, $m_\eta =0.547853$, $F_\pi =0.09221$.

3 The inelastic mode $\eta \pi \rightarrow 3\pi$ is allowed already at threshold but the *S*-wave projection vanishes by parity conservation (since $J^P=0^-$ for the $3\pi$ state). The modes $\eta \pi \rightarrow 5\pi$, $\eta \pi \rightarrow \eta 3\pi$ are strongly suppressed by phase space below 1 GeV.

4 Equality of the two cross sections occurs either when $\eta =0$ or $\eta =\cos 2\delta _{11}$.
